# Successful reconstruction using three flaps combination after wide excision due to basosquamous carcinoma: A case report

**DOI:** 10.1016/j.ijscr.2023.109150

**Published:** 2023-12-11

**Authors:** Sedra Sheikh Debs, Maher Badawi, Ghina Majd Hussain, Yazan Soliman Khadour, M.Noor Khouja, Aladdin Etr

**Affiliations:** a Faculty of Medicine, University of Aleppo, Aleppo, Syria; b Faculty of Medicine, Tishreen University, Lattakia, Syria; c Faculty of Medicine, Tartous University, Tartous, Syria; d Department of Plastic Surgery, Faculty of Medicine, Aleppo University Hospital, University of Aleppo, Aleppo, Syria

**Keywords:** Basosquamous carcinoma, McGregor flap, V-Y glabellar flap, Nasolabial laterally based cheek flap, Case report

## Abstract

**Introduction:**

Basosquamous carcinoma (BSC) is an uncommon and malignant subtype of non-melanoma skin cancer. It has features that are halfway between basal cell carcinoma (BCC) and squamous cell carcinoma (SCC).

**Case presentation:**

An 87-year-old female presented with a lesion on her left cheek, nasal ala, medial canthal area, and eyelids. After investigations, which included biopsies and a computed tomography scan (CT), surgery was decided upon to completely remove the mass. A 5 × 4 cm defect after the surgery was reconstructed by mobilizing three flaps, including the McGregor flap, V—Y glabellar flap, and nasolabial laterally based cheek flap. The excisional biopsy detected malignancy at the lateral border of the upper lid, which led to the patient undergoing surgery to remove the tumor formation. After the second surgery, the histopathology confirmed no malignancy. The patient had functionally and aesthetically pleasing results, preserved eyelid movement and visual field. No surgical complications or recurrences occurred within the first year after the surgery.

**Discussion:**

BSC is a neoplasm without well-defined histologic characteristics or standardized treatment procedures compared to other non-melanoma skin cancers. However, several studies recommend using wide excision. In our case, Reconstructing the area was challenging due to important anatomical structures and finding tissue that matches the desired appearance, while preserving functional and aesthetic results.

**Conclusion:**

In this case report, we highlight the value of reconstructing face defects after wide excision due to BSC using three flaps.

## Highlights

• Basosquamous carcinoma is a rare and aggressive subtype of non-melanoma skin cancer.
• Wide excision and reconstruction are effective to treat basosquamous carcinoma.
• Combining three flaps successfully repaired the wide excision of 4 × 5 cm in patient's face.
• The reconstruction must maintain a natural look, symmetry, visual field, and eyelid movements.

## 1 Introduction

Basosquamous carcinoma is a rare and aggressive subtype of non-melanoma skin cancer, displaying characteristics intermediate between basal cell carcinoma (BCC) and squamous cell carcinoma (SCC) [1], and constitutes approximately 2 % of all non-melanoma skin cancer cases [2].

Basosquamous carcinoma was initially identified by Mac-Cormac in 1910 as a histological variation within a series of rodent ulcers, where basal cell and squamous cell tumors were present adjacent without a transition zone [3]. As a result, this malignancy is regarded as an especially aggressive form of BCC, exhibiting heightened risks of metastasis and recurrence compared to typical BCC cases [3].

The clinical and histologic morphology of BSC is variant and atypical, making it a diagnostic and treatment challenge. In addition, the biological behavior of BSC is unpredictable [1].

Presently, no established clinical guidelines exist for the management of basosquamous carcinoma cases [4]. Yet several studies recommend employing wide excision [5].

In this case, we describe a successful surgical approach employing three flaps to reconstruct the wide excision in an 87-year-old female patient due to basosquamous carcinoma affecting the left cheek, nasal skin and eyelids. This manuscript was prepared by the SCARE 2020 guidelines [6].

## 2 Presentation of case

**Fig. 1:**
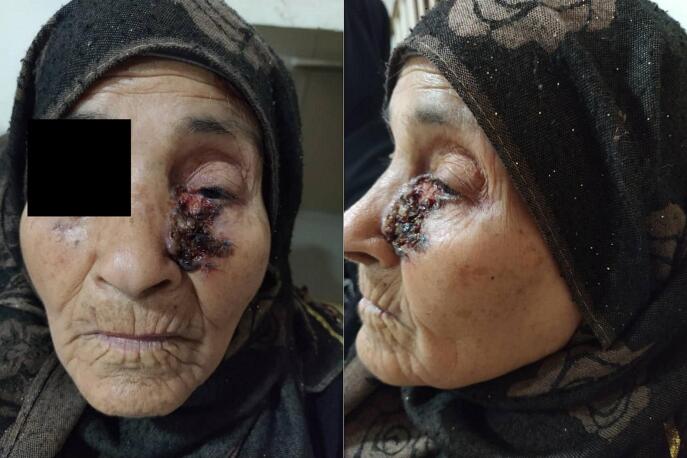
The lesion on the patient's face, extending on left cheek, nasal ala, medial canthal and the eyelids.

We report a case of an 87-year-old female patient who presented with a lesion on her left cheek, nasal ala, medial canthal area, and eyelids to the hospital (Fig. 1). There were no known precipitating factors, and the patient had no significant medical history.

Two small, irregular, non-orientable grayish cutaneous biopsies, measuring 0.8 cm and 0.6 cm, were submitted for microscopic examination. The examination of 16 different levels of sections revealed a malignant, infiltrative proliferation of predominantly basaloid cells with focal squamous differentiation, suggesting a differential diagnosis including Basosquamous Carcinoma (BSC) and malignant hidradenoma. Furthermore, an inflammatory infiltrate rich in plasma cells, lymphocytes, and abundant melanophages was observed. The biopsy specimens did not show any free margins.

A CT scan was performed, revealing a 22 × 6 mm mass with ragged edges at the level of the left cheek skin, extending to the nasal skin. The mass did not infiltrate adjacent bones or underlying adipose tissue, and no evidence of bone metastases at the level of the skull was observed. There was no lymphadenopathy seen in the neck or abdomen, nor around the blood vessels in these areas. However, mild lymphadenopathy was observed in the mediastinal lymph node, with the largest measuring 1.5 cm. Fluid density was noted in both the right and left maxillary sinuses, while no free fluid was observed in the abdomen or pelvis.

**Fig. 2:**
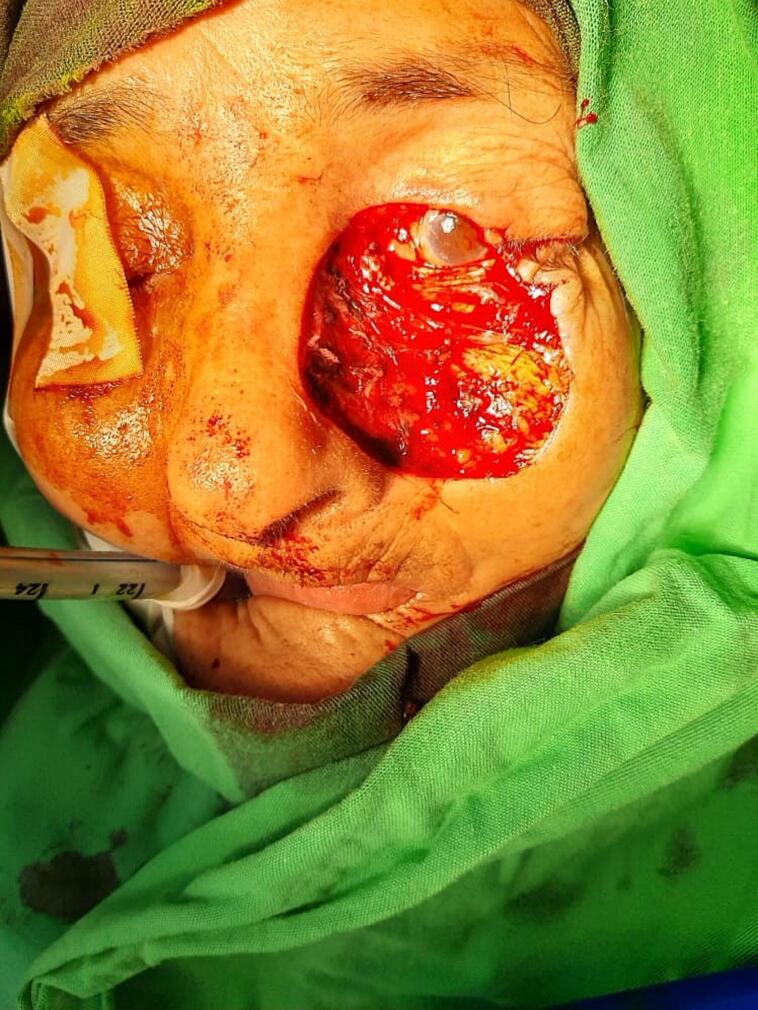
The wide excision during the surgery.

**Fig. 3:**
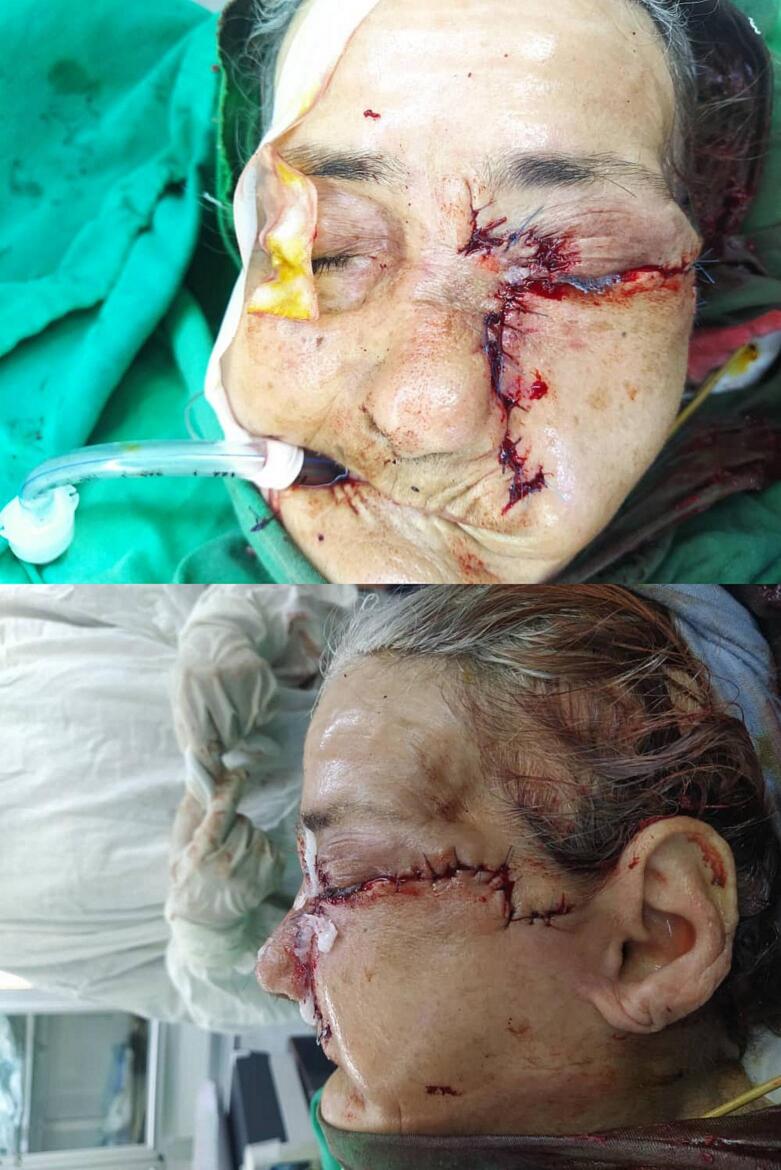
The stitches after the surgery.

Based on these previous findings, the patient had to undergo surgery to remove the mass. A wide excision was performed, which involved the removal of nearly 90 % of the lower eyelid, half of the upper eyelid, the medial canthus, and a portion of the nose and left cheek (Fig. 2). The procedure was performed under general anesthesia. During the procedure, the surgical team encountered some difficulty due to the proximity of the tumor to the eye and the complex anatomy of the facial region. However, with careful planning and execution, the team was able to achieve tumor removal while preserving the patient's visual function. Reconstructive surgery was also performed using three skin flaps: the McGregor flap, the V—Y glabellar flap, and the nasolabial lateral cheek flap. The surgical team carefully planned the reconstruction process to achieve good cosmetic and functional outcomes (Fig. 3).

**Fig. 4:**
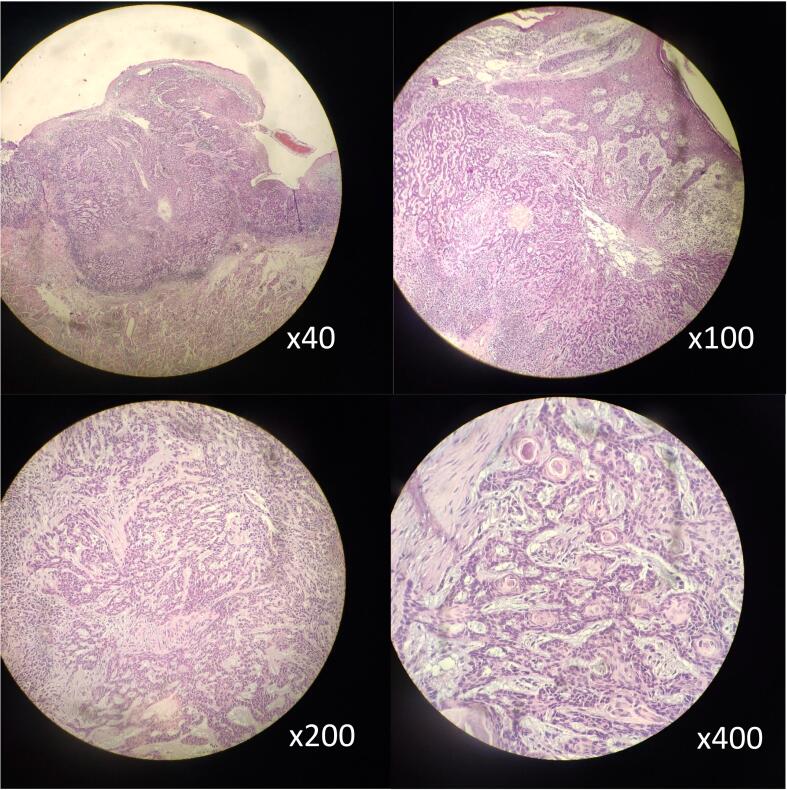
Basosquamous carcinoma, basaloid and squamous cells, Hematoxylin-Eosin stain; original magnifications: ×40, ×100, ×200, ×400.

The excisional biopsy, taken from the mass after the surgery, confirmed the diagnosis of Basosquamous carcinoma. The biopsy showed epithelial proliferation within the dermis, consisting of basaloid cells with large oval nuclei and little cytoplasm, and with the presence of focal peripheral palisades. The surrounding stroma had shrunk to form narrow clefts, and other sections showed nests of malignant squamous cells surrounded by dense desmoplastic stroma (Fig. 4).

The report indicated that all the lateral and deep borders were free of malignancy, except for a lateral border in the upper eyelid, which was not free of malignancy.

Following the report, the patient underwent surgery to remove the remaining malignancy that was detected in the lateral border of the upper eyelid three months prior.

The Incisional biopsy of the upper eyelid (measuring 0.8 × 0.6 × 0.5 cm) showed mild hyperkeratosis and a moderate perivascular mononuclear inflammatory infiltrate, as well as thick collagen bundles. However, the biopsy was found to be within normal limits and no malignancy was detected.

The patient was referred to the oncology department and no adjuvant therapies were added.

**Fig. 5:**
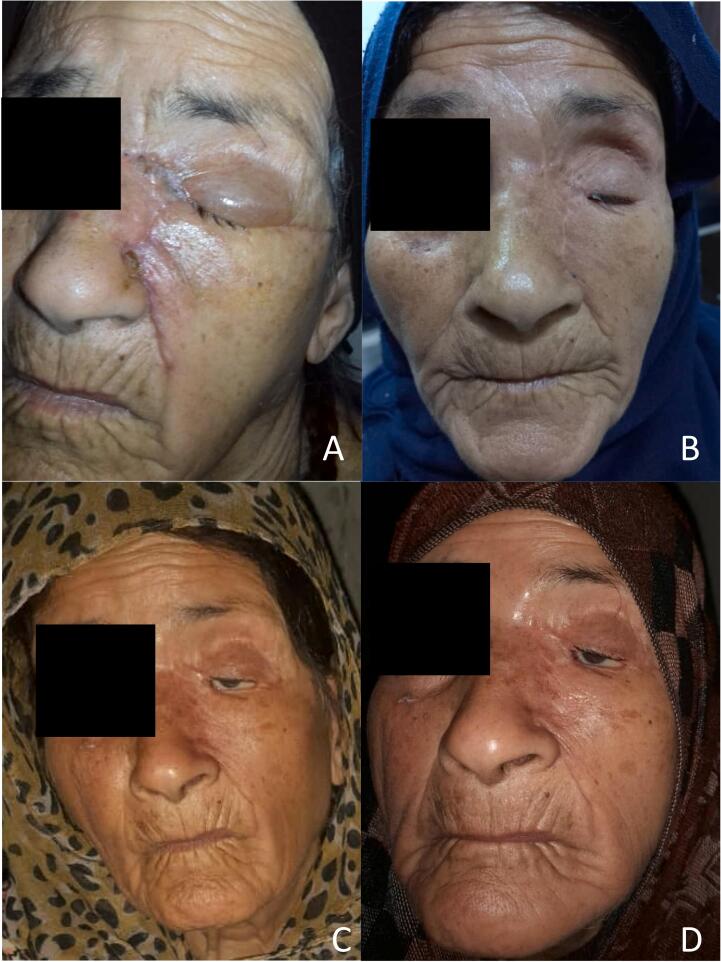
A: two weeks after the first surgery. B: three months after the first surgery. C: five months after the two surgeries. D: one year after the two surgeries.

The patient was in good health after the two surgeries and regained the ability to close the eyelid despite the procedures. The patient's visual acuity was not affected, and there were no surgical complications or recurrences within one year of the surgery (Fig. 5).

## 3 Discussion

BSC is a malignant and aggressive neoplasm without well-defined histologic characteristics or standardized treatment procedures compared to other non-melanoma skin cancers. However, several studies recommend using wide excision [5]. This cancer most commonly occurs in sun-exposed parts of the head and neck (82–97 %), particularly the ears and perinasal region. However, the trunk and extremities also showed BSCs, although comparatively fewer [1]. High UVR exposure and Fitzpatrick types I–II are regarded as significant risk factors for the development of a BSC. The tumor typically manifests in elderly patients, with a significant male preponderance (patients over 70 years old account for 34.4 % of all BSC cases) [1].

The best diagnostic method for BSC is still a biopsy and histologic study, which indicates the presence of a transition zone between the BCC and SCC histologic features [1]. The incidence of recurrence and risk of metastasis are considered to be higher in BSC when compared to other types of BCC. Recurrence rates of 10 % to 48 % have been reported, and the metastatic rate may range from 5 % to 7.4 % [1]. Male sex, positive surgical margins, lymphatic invasion, and perineural invasion were found to be the most significant predictors of topical recurrence for BSCs [1].

In our case, the defect sizes after wide excision were 5 × 4 cm. The defect was too wide to be covered with a single flap, which suggested a combination of flaps. We decided to combine the McGregor flap with the V—Y glabellar flap and the nasolabial laterally based cheek flap. The results were both functionally and aesthetically pleasing.

An orbicularis oculi myocutaneous flap could be applied to repair small defects, especially in the elderly with extra skin on the upper eyelid. But wide defects cannot be reconstructed using this flap. Also, this flap is not suggested in younger patients who lack extra skin in the area of the upper eyelids [7].

A bilobed flap, which is lifted from the nasal dorsum, is an effective option for tissue harmony and color, but it is also unable to cover wide defects [7].

We did not consider reconstructing using grafts because it can cause a difference in skin tone and texture, unlike local flaps.

Additionally, A graft could be applied to reconstruct defects less than 2 cm usually [8].

Reconstructing the area poses a difficult challenge due to critical anatomical structures. Also finding tissue that matches the desired color and thickness is challenging. The reconstruction must ensure the maintenance of the area's natural appearance without causing any deformation to the surrounding tissues, establish symmetry, and preserve the visual field and eyelid movements.

Lower lid surgery frequently results in epiphora, especially in the early postoperative days.

But careful patient assessment, accurate surgical technique, and suitable postoperative care reduce the risk of complications following eyelid surgery.

Other complications after eyelid surgeries include suture granuloma, infection, eyelid hematoma.

In our case the patient was followed up for one year and there were no complications [9].

After the surgery, the patient did not receive adjuvant therapies, due to her old age and nonexistence of metastasis.

Hiromichi Matsuda et al. showed in 2014 that a nasolabial V—Y advancement flap and a glabellar subcutaneous pedicled flap successfully reconstructed a 7 × 11 mm defect in the medial canthal region, resulting in a good cosmetic result [10].

## 4 Conclusion

Combining McGregor flap, V—Y glabellar flap, and nasolabial laterally based cheek flap successfully repaired the wide defect including nearly 90 % of the lower eyelid, half of the upper eyelid, the medial canthus, and a portion of the nose and left cheek. This approach preserved eyelid movements and enabled full closure. The cosmetic results were good.

## Abbreviations

BSC: Basosquamous Carcinoma
BCC: Basal cell carcinoma
SCC: Squamous cell carcinoma
CT: Computed tomography

## Ethical approval

Single case reports are exempt from ethical approval in our institution, as the paper does not contain any information that identify the patient. Moreover, consent was taken from the patient.

## Funding

This research did not receive any specific grant from funding agencies in the public, commercial, or not-for-profit sectors.

## Author contribution

SS & MB & GH & YK & MNK: Writing - Original Draft and editing.

AE: supervision, manuscript review and the plastic surgeon.

All authors have read and agreed to the published version of the manuscript.

## Guarantor

Sedra Sheikh Debs, Maher Badawi.

## Research registration number

N/A

## Consent

Written informed consent was obtained from the patient for publication of this case report and accompanying images. A copy of the written consent is available for review by the Editor-in-Chief of this journal on request.

## Conflict of interest statement

The authors declare no conflict of interest.

## Data Availability

All data on which the conclusions of this case report are based are included in this manuscript.
